# Leishmaniasis sand fly vector density reduction is less marked in destitute housing after insecticide thermal fogging

**DOI:** 10.1186/1756-3305-6-164

**Published:** 2013-06-06

**Authors:** Luis Fernando Chaves, Jose E Calzada, Chystrie Rigg, Anayansi Valderrama, Nicole L Gottdenker, Azael Saldaña

**Affiliations:** 1 Programa de Investigación en Enfermedades Tropicales (PIET), Escuela de Medicina Veterinaria, Universidad Nacional, Heredia, Costa Rica; 2 Institute of Tropical Medicine (NEKKEN), Nagasaki University, 1-12-4 Sakamoto, Nagasaki, 852-8523, Japan; 3 Departamento de Parasitología, Instituto Conmemorativo Gorgas de Estudios de la Salud, Ciudad de Panamá, República de Panamá; 4 Departamento de Entomología, Instituto Conmemorativo Gorgas de Estudios de la Salud, Ciudad de Panamá, República de Panamá; 5 Department of Pathology, School of Veterinary Medicine, University of Georgia, Athens, GA, USA

**Keywords:** Panama, *Lutzomyia gomezi*, *Lu. trapidoi*, Deltamethrin, Housing quality

## Abstract

**Background:**

Insecticide thermal fogging (ITF) is a tool to control vector borne diseases. Insecticide application success for vector control has been associated with housing materials and architecture. Vector abundance is correlated with weather changes. Nevertheless, housing quality and weather impacts on vector abundance have been unaccounted for in most New World insecticide control trials for leishmaniasis vectors.

**Methods:**

We conducted a 15 month insecticide control trial that included two deltamethrin [6 mg a.i.m^-2^] based ITF interventions in 12 of 24 monitored houses at Trinidad de Las Minas, a hyperendemic cutaneous leishmaniasis transmission village in western Panamá. During the study we followed sand fly (SF) abundance, keeping track of rainfall and quantified housing quality using an index based on architecture and construction materials.

**Results:**

We found a 50 to 80% reduction in SF density in the fogged houses when compared with control houses, while controlling for seasonal changes in SF abundance associated with rainfall. We found heterogeneities in the reductions, as abundance changed according to SF species: *Lutzomyia gomezi, Lu. panamensis, Lu. dysponeta* and *Lu. triramula* reduced in density between 40% and 90% after ITF. In contrast, *Lu. trapidoi* density increased 5% after ITF. Differences in the impact of ITF were associated with housing quality, the most destitute houses, i.e., those with features that ease insect entrance, had a disproportionally larger SF abundance, in some cases with increased domiciliary SF density following the ITF.

**Conclusion:**

Our results suggest the potential of insecticide application to control SF density and leishmaniasis transmission could depend on housing quality beyond insecticide efficiency.

## Background

Cutaneous Leishmaniasis (CL) is a major neglected tropical disease worldwide [1,2]. In relative terms, CL in the new world remains a poorly studied disease [2,3], as are Sand Fly (SF) vectors of *Leishmania* spp parasites [4-6]. For example, studies on SF ecology and control are somewhat scarce [5-7] when compared with other vectors of pathogens, especially mosquitoes [8-10]. Long-term studies looking at the population dynamics of SF vectors [11-18], and studies on insecticide based control of SF abundance [7] are limited. Insecticide control trials for New World sand flies have been carried out using organophosphates: Malathion [19]; organochlorines: DDT [20,21] and pyrethroids: cyfluthrin [22], cypermethrin [23,24], deltamethrin [25-30], lambdacyhalothrin [31-33]. Methods of insecticide application for SF control have included: fogging [19,22,34], residual spraying [21,23-27,31-33], insecticide diffusion devices [35], insecticide treated nets [28,29] and curtains [30]. In general, these studies have shown that insecticide application at the household level seems to be the most effective SF control method [7], because it can suppress both SF abundance [7] and CL transmission over short [36] and long term [37] time scales. However, a key insight from the few long-term studies on SF population dynamics is that vector abundance is extremely sensitive to environmental changes, with different species having a distinctive sensitivity to particular meteorological components [12,15,17]. Nevertheless, most insecticide control trials have ignored the importance of weather variability when evaluating the efficacy of insecticides on SF abundance [20-35].

CL has also been recognized as a disease of poverty [38,39]. For example, our work has shown that in Central America CL primarily affects socially excluded populations, i.e., those who lack or have restricted access to resources that ensure a satisfactory quality of life [40,41]. This fact is extremely important to better understand the dynamics of disease transmission. Because CL transmission is ruled by ecological processes [42], how humans become part of a pathogen’s life cycle, especially in neglected tropical diseases like leishmaniasis, can be heavily influenced by social factors [43]. Even if recognized as an important factor for the success of insecticide applications in CL control [23,32], housing quality has been unaccounted for when evaluating the outcome of insecticide trials.

In Panamá and Costa Rica, clinical treatment of patients with skin lesions is the main activity pursued to control CL transmission [44,45], neglecting active surveillance and SF vector control. Nevertheless, the disease is becoming increasingly common in both countries. For example, a recent urban CL outbreak occurred in Tilarán, Guanacaste, Costa Rica [44]. Similarly, a CL epidemic in Western Panamá province, Panamá, where 500 new CL cases (~50% of them in children < 5 years of age) were officially reported, representing a two-fold increase for this area when compared with recent years [45]. These outbreaks suggest that changes are taking place in CL transmission epidemiology in Central America, calling for the implementation of vector control as a major strategy to reduce the impact of CL on populations at risk of acquiring the disease [7,41]. Here, we present results from a small scale SF insecticide control trial carried out at Trinidad de Las Minas, Western Panamá Province, República de Panamá. Our goal was to evaluate the impact of household insecticide thermal fogging (ITF) with deltamethrin (6 mg a.i.m^-2^) on domiciliary and peridomiciliary SF abundance. Baseline observations showed that household human infection rates were positively associated with the household abundance of *Lutzomyia gomezi*, but also with *Lu. panamenesis* and *Lu. trapidoi*[45], the most abundant vector species in the area [46]. For 15 months we followed domiciliary and peridomiciliary SF abundance in 24 houses, 12 were subjected to two rounds, 6 months apart in time, of Insecticide Thermal Fogging (ITF) with deltamethrin at 6 mg a.i.m^-2^ and 12 kept as control. We found a 50 to 80% reduction in SF density in the fogged houses when compared with control houses, while controlling for seasonal changes in SF abundance associated with rainfall. We observed a variety of species specific abundance changes, with *Lu. gomezi, Lu. panamensis, Lu. dysponeta* and *Lu. triramula* reducing their density between 40% and 90% after ITF, in contrast to *Lu. trapidoi* whose density increased 5% after the ITF. Spatially, we found that heterogeneities in SF abundance after ITF were associated with housing quality, specifically destitute houses, i.e., those with features that ease insect entrance, had the largest share of SF individuals, in some cases with an increased domiciliary SF density following the ITF. Thus, our results call for a better quantification and understanding of housing quality as a major factor underpinning the success of insecticide control for leishmaniasis vectors.

## Methods

### Study Area

Our study was conducted at Trinidad de Las Minas, (8°46’32‘N and 79°59’45‘W), a rural village in Capira District, western Panamá Province, República de Panamá. This village is 230 meters above sea level, with an annual mean temperature of 26.0°C and monthly rainfall ranging from 28 — 570 mm^3^. Climate is markedly seasonal, with a dry season from mid December to March and a rainy season for the rest of the year. The area used to be a lowland tropical moist forest, but currently is a transitional forest/agricultural matrix, with scattered deciduous and xerophile species. Further details about the study site are presented by Calzada *et al.*[46]. Daily rainfall records for our study period were obtained from a meteorological station within a 5 Km radius from our study site, managed by Panamá’s electrical company ETESA.

### Insecticide thermal fogging

We selected 24 houses for our study (out of 128 houses in the village), where residents provided informed consent to collect sand flies inside and outside their houses. Twelve houses were subjected to indoor and outdoor insecticide thermal fogging, while the remaining 12 houses were kept as controls (no fogging). The number of houses evaluated in this study was primarily limited by the availability of sampling resources, especially the number of light traps. Nevertheless, based on the only previous study on SF control by fogging in Panamá [19] a sample of 12 houses for the insecticide treatment and 12 control houses is powerful enough (1-β > 0.80, given α < 0.05) to detect differences in sand fly abundance due to insecticide thermal fogging (ITF), using a generalized linear model with one treatment and one covariate (See Additional file 1: Protocol S1 and Additional file 2: Figure S1). We selected a cluster of houses with homogeneous eco-epidemiological conditions and where intra-domiciliary SF presence was confirmed by residents. Although we planned to match houses based on construction materials and architecture, our selection was limited by the lack of informed consent from some residents for the fogging, especially in the houses with the best construction materials. However, to counter this limitation, we carefully recorded details about housing construction and the peridomicile and vegetation of each house (see section below on *Housing destituteness, the peridomiciliary environment, peridomiciliary vegetation structure and animal abundance/richness assessment*) that were considered during the statistical analysis. We evaluated two rounds of indoor/outdoor ITF using deltamethrin (K-Othrine® 2.7 UBV, Bayer, Guatemala). Insecticide selection and application was performed by trained personnel of the Vector Control Department from Panamá´s Ministry of Health. For Insecticide selection, results from toxicity assays in naïve populations were considered [32,47-49], especially given that our study site has never been subjected to deltamethrin application for vector control. It is also worth highlighting that agriculture is primarily organic at Trinidad de Las Minas. The insecticide applications were conducted on July 18, 2010 and January 23, 2011. The insecticide was applied with a hand-held thermal fogger (Golden EagleTM, Model # 2610, Curtis Dyna-Fog Ltd, Westfield, IN, USA) to interior and exterior housing walls, targeting cracks and crevices. A similar fogging was performed in the 15 m around the houses (peridomicile). We choose the 15 m radius for the fogging based on studies on New World SF dispersal in which SF rarely travel beyond 50 m from a release point [50-52]. On average, 0.57 L of insecticide (diluted in diesel to a final concentration of 0.7 g /L, following Panamá Ministry of Health guidelines) was used for the fogging of internal and external house walls (whose total wall surface on average was 65 m^2^), corresponding to a concentration of 6 mg of active ingredient per square meter (mg a.i.m^-2^).

### Ethical clearance

This study was approved by the National Review Board, Comité Nacional de Bioética de la Investigación, Instituto Conmemorativo Gorgas de Estudios de la Salud, Ciudad de Panamá, República de Panamá (561 /CNBI/ICGES/06).

### *Sand fly* (SF) *abundance*

We evaluated ITF impacts on SF abundance by comparing collections from the domicile and peridomicile of fogged and control houses. Sand flies were collected using modified light-traps [53]. Each trap was slightly modified by attaching an additional small LED light to increase SF attraction [46]. Entomological sampling was carried out monthly from April 2010 to June 2011, except for the months of August and November 2010 and January 2011, when access to this remote village was impossible because of logistical and operational constraints, which, in January 2011, prevented the sampling of houses just before the 2nd ITF. Thus, a total of 12 sampling surveys were conducted during the study. For each monthly collection, one trap was placed for one night in the main bedroom of every household (indoor). This trap was suspended from the ceiling at about 2 m from the ground floor. Another trap was placed at the same height, above vegetation, within 50 meters of the house (i.e., peridomicile). Traps were setup for 12 hours, from 6:00 pm to 6:00 am, in the same position (indoor and peridomicile) during each sampling session.

Trapped sand flies were removed from the traps, stored at −20°C to kill the insects and preserved in 70% ethanol for identification. For each trap, we summarized the abundance, sex and species of sand flies following Young and Duncan [54], with male genitalia and female spermathecae as main diagnostic taxonomic characters. A detailed description of the SF fauna species composition at Trinidad de Las Minas and changes following the ITF is presented by Calzada *et al.*[46].

### Housing destituteness, the peridomiciliary environment, peridomiciliary vegetation structure and animal abundance/richness assessment

For each house, we collected data on construction materials and the presence of insect friendly gaps to construct an index of house quality, hereafter referred to as housing destituteness index, HP. We specifically collected data on materials used for walls and roofs, whether the floor was earthen or covered with concrete or wood. We recorded the presence of insect friendly gaps, i.e.; whether walls had crevices, if walls were complete from the base to the roof, whether doors had holes and if the houses had windows and whether the windows had anti-insect screening. We recorded the presence/absence of elements that may serve as peridomicilary resting places for sand flies, such as rubbish, fruit trees, etc. [12,55] to estimate a peridomicile index (PI). We measured several elements of the vegetation structure in the peridomicile for the estimation of a vegetation index (VI). To quantify any possible role that vertebrate host abundance could have on sand fly density, we performed a census on all domestic animals belonging to each household and compiled a list of wildlife species seen by household residents in the domiciliary/peridomiciliary area of each household. We used these data to estimate animal abundance indices. HP, PI, VI and the animal abundance indices were estimated by computing the first principal component for the set of variables considered in each index, for further details about the variables considered for each index, and the principal components analysis implementation see Additional file 3: Protocol S2. For both domestic and wild animals we also estimated species richness at each household by, respectively, counting the number of domestic species, recorded in the domestic animal census, or the number of reported wildlife species seen by the householders.

### Statistical analysis

For the analysis we employed a two-fold strategy, we evaluated the impact of the fogging both temporally and spatially. For the temporal analysis we employed negative binomial generalized linear models (NB-GLM) that accounted for overdispersion in SF counts [56]. As a first approach, models considered all SF species abundance, separating groups according to the feeding habit i.e., whether sand flies have been recorded biting humans or not (i.e., anthropophilic or zoophilic) [42], and sampling habitat (peridomicile and domicile). We also considered the monthly average and the standard deviation, S.D., of daily rainfall, with a one month lag, the lag selected with a cross-correlation analysis [57], as a covariate to account for seasonal abundance fluctuations in SF abundance and whether a house was fogged or not. We included rainfall S.D. as covariate to account for rainfall variability as an underpinning factor of SF abundance, specifically SF abundance could be sensitive not only to the amount of rain during a time period, a quantity measured by the average rainfall, but also to the intensity of the rainfall events [12], a quantity measured by the S.D. For the insecticide we considered whether fogging independently of the application date had an impact on SF density (Fogging A in the models) or whether the fogging impact was different for each of the two fogging rounds (Fogging B in the models). We further developed models for the three main dominant vectors in our study site: *Lu. gomezi*, *Lu. panamensis* and *Lu. trapidoi* (which accounted for ~60% of the collected sand flies, Table 1 and [46]) and for the two most abundant non vector-species *Lu. triramula* and *Lu. dysponeta* (Table 1); comparing temporal dynamics in the domicile and peridomicile of fogged and control houses. For the spatial analysis we also employed NB-GLMs. In these models we looked at the cumulative number of sand flies caught after the foggings as a function of housing destituteness (HP), the peridomiciliary environment index (PI), the vegetation structure index (VI) and the animal abundance/richness indices. We considered possible non-linearities in the association of HP, PI, VI and the animal abundance/richness indices with SF abundance by fitting polynomials and models with breakpoints [40], i.e., threshold values in the independent variables at which the association with a dependent variable can change quantitatively, employing hockey stick regressions (see Additional file 1: Protocol S1 for further details). For both the temporal and spatial models we performed model selection based on the Akaike Information Criterion, a tool for model selection that guides the choice of a “best model” based on its likelihood to explain the data with a minimum number of parameters [56]. For the best temporal model, beyond the assumption of the NB-GLM, we tested the temporal independence of the residuals (error) through the inspection of the autocorrelation function, ACF, and the partial autocorrelation function, PACF [57]. For the best spatial model we tested the spatial independence of the residuals with the Moran’s I test.

**Table 1:** Sand Fly species abundance in the control and fogged houses

**Species**	**Control**		**Fogged**	
	**Domicile**	**Peridomicile**	**Domicile**	**Peridomicile**
*Lutzomyia trapidoi* (Fairchild & Hertig)	562	158	228	203
*Lu. gomezi* (Nitzulescu)	448	238	291	169
*Lu. panamensis* (Shannon)	99	470	88	310
*Lu. triramula* (Fairchild & Hertig)	71	902	25	152
*Lu. dysponeta* (Fairchild & Hertig)	67	193	126	104
Other anthropophilic species	148	79	68	76
Other zoophilic species	87	116	51	88
Unidentified	2	1	4	4
**Total**	1484	2157	881	1106

The bottom row shows the total number of individuals sampled in each category. A total of 24 houses were monitored (12 as control and 12 for the fogging) and each house underwent a total sampling effort of 24 trap nights (12 Domicile and 12 Peridomicile). Abundance is for combined male and female counts.

## Results

### Sand fly fauna description

We collected 5628 sand flies in the 576 sampling night-traps (Table 1). We were unable to identify 11 individuals, all other individuals belonged to 24 SF species (full details have been published elsewhere [46]). Of the collected species, 8 had a proven vector status [41,42] and 3 accounted for more than 50% of the samples: *Lu. trapidoi* (20%) *Lu. gomezi* (20%) and *Lu. panamensis* (17%). Of the remaining SF species, *Lu. triramula* (20%) and *Lu. dysponeta* (8.7%) were the most abundant. Sand flies were more abundant outside (peridomicile) than inside (domicile) the studied houses (58% vs 42%). Table 1 also shows that total abundance of sand flies was reduced by approximately 40% inside the fogged houses (i.e., domicile; control: 1484, fogged: 881) and close to 50% in the peridomiciliary environments (control: 2157, fogged: 1106). Further details are presented by Calzada *et al*. [46]

### Temporal impacts of insecticide thermal fogging on all Sand Fly species

**Figure 1:**
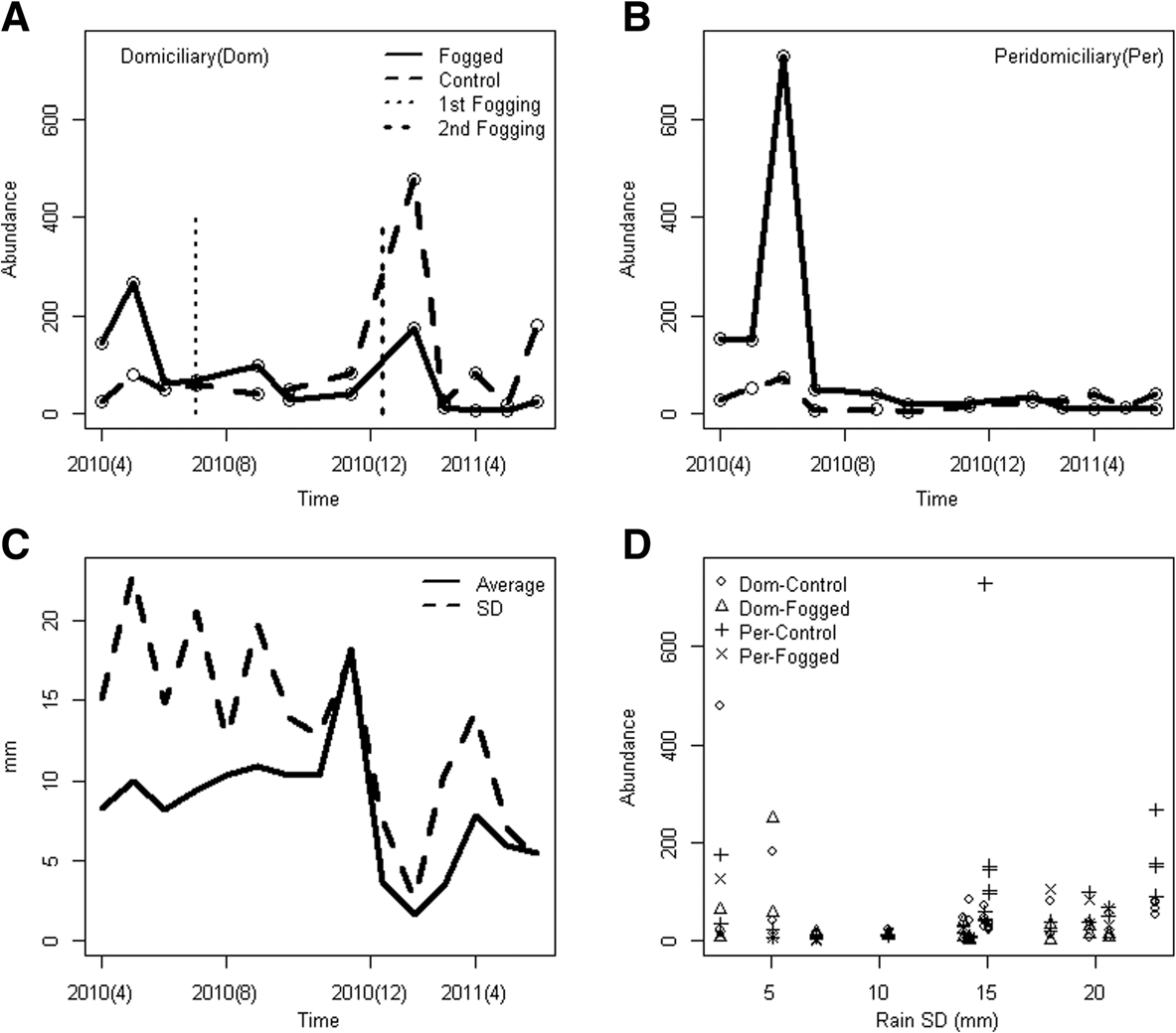
**Vector Abundance and Rainfall.** (**A**) Domiciliary (Dom) vector species abundance, vertical lines indicate the timing of the Foggings (**B**) Peridomiciliary (Per) vector species abundance (**C**) Monthly Rainfall, daily mean and S.D. (**D**) Sand Fly abundance as a function of monthly S.D. of daily Rainfall records, symbols are used to separate domiciliary and peridomiciliary species from control and fogged houses. In (**A**) and (**B**) the open circles indicate the measured values, where each point represents the cumulative abundance from the 12 trap-nights per treatment.

Additional file 4: Table S1 shows the different models considered to explain the population dynamics of all the SF species in the domicile (Figure 1A) and peridomicile (Figure 1B). Rainfall (Figure 1C) seemed to be an important factor shaping SF population dynamics, so was insecticide fogging (Figure 1D). The best model found statistically significant differences in SF abundance according to vectorial status (vector, i.e., anthropophilic or non-vector, i.e., zoophilic), environment (domicile and peridomicile), rainfall (a second degree polynomial), fogged vs control houses, as well as the interaction of vectorial status with the fogging and the environment (Additional file 4: Table S1). Quantitatively, the best model (Table 2) indicates that non-vector (zoophilic) sand flies were most abundant in the peridomiciliary environments (on average 308/per house and sampling night), followed by vector (anthropophilic) SF species (about 22% less individuals than non-vectors in the domicile). Inside the houses (domicile environment), vector species (anthropophilic) were the most abundant (about 78% of the zoophilic sand flies observed in the peridomicile). Fogging reduced the abundance of vector sand flies inside the houses by 51%, and 77% in the peridomicile (when compared with non-vector species in the peridomicile). Rainfall variability, measured by Rainfall S.D., had a concave relationship with SF abundance, i.e., a second degree polynominal with a minimum, in other words SF abundance was higher at low and high levels of rainfall variability, a feature that could reflect the different responses of SF to environmental variability. As suggested by Chaniotis *et al.*[12] some sand flies might become very abundant when rainfall is sustained, while others might thrive when there is a high alternancy of dry and wet periods. All the assumptions of the GLM model were not violated and model residuals were not autocorrelated. In general, all the estimated parameters accurately described the patterns observed in Figure 1.

**Table 2:** Parameter estimates for a negative binomial model explaining the abundance of all Phlebotomine sand fly species in Trinidad de las Minas, Capira District, Panamá

**Parameter**	**Proportional abundance change**	**Estimate**	**S.E.**	***z***	**Pr(>|z|)**
Control-Peridomicile-Zoophilic	1(308)^¶^	5.730	0.451	12.712	<0.00001^x^
Domicile	0.416	−0.878	0.268	−3.274	0.00106^x^
Anthropophilic	0.783	−0.245	0.302	−0.812	0.417
Fogged	0.228	−1.477	0.282	−5.246	<0.00001^x^
(S.D. Rain)^2^	1.008	0.00875	0.00265	3.301	0.000964^x^
S.D. Rain	0.795	−0.230	0.0689	−3.338	0.000844^x^
Domicile*Anthropophilic	0.784^§^	0.880	0.377	2.335	0.0195^x^
Fogged*Anthropophilic	0.482^§^	0.993	0.391	2.540	0.0110^x^

^x^Statistically Significant (P < 0.05). ^¶^the value inside parenthesis is the estimated abundance for the reference group, i.e., Control-Peridomicile-Zoophilic. * indicates a synergistic (a.k.a. interaction) effect. ^§^To estimate the proportional abundance change of these interactions we considered the value in relation to the estimate for the main factors.

The overdispersion parameter estimate (± S.E.) was 1.217 ± 0.164. The model considered whether a house was fogged or not (Control), the habitat (Domicile or Peridomicile), whether species are known to feed on humans (Anthropophilic) or not (Zoophilic) and a second degree polynomial for the monthly S.D. of daily Rainfall.

### Temporal impacts of insecticide thermal fogging on the most abundant Sand Fly species

**Figure 2:**
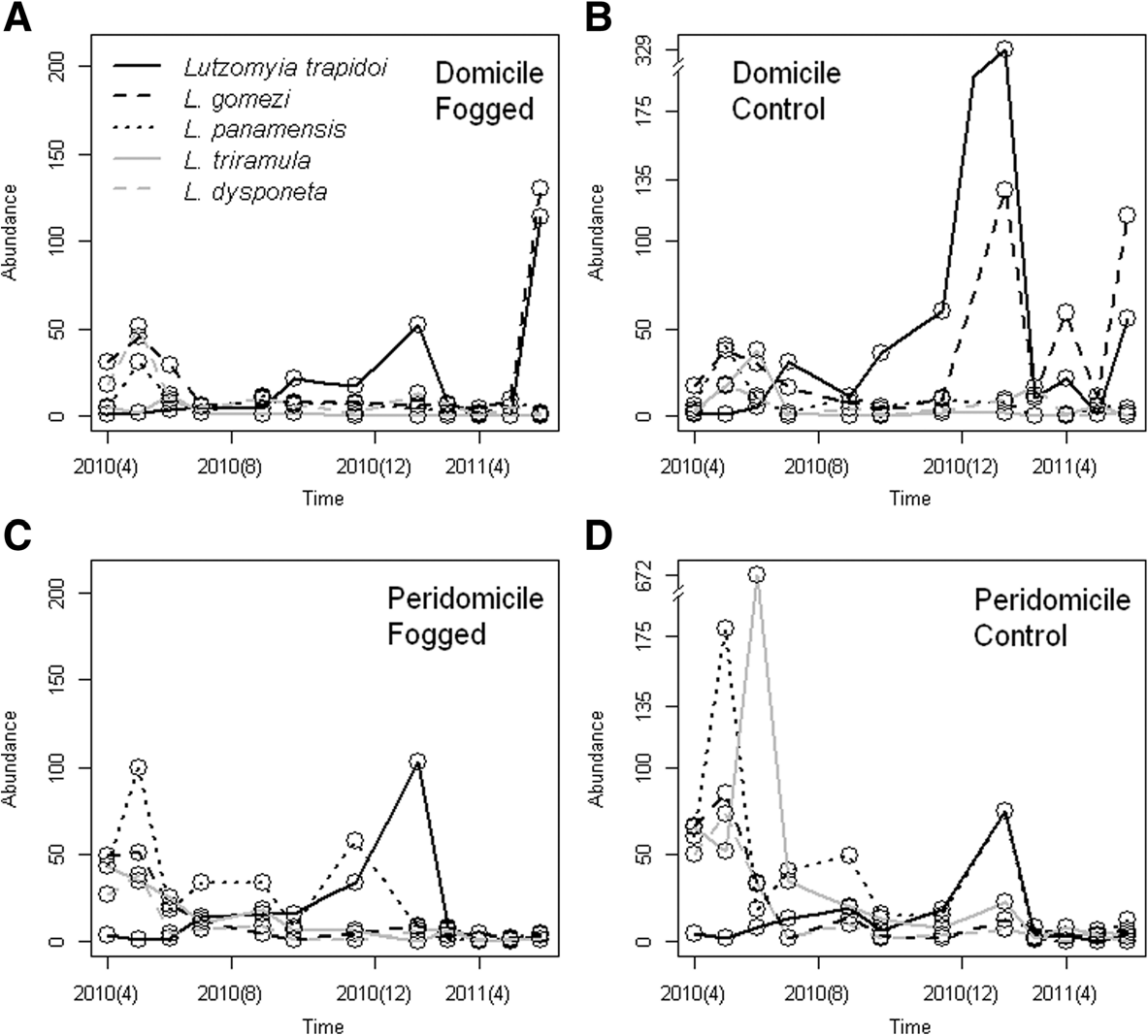
**Common species abundance patterns.** (**A**) Domiciliary environment of fogged houses (**B**) Domicilary environment of control houses (**C**) Peridomiciliary environment of fogged houses (**D**) Peridomicilary environment of control houses. In the panels open circles indicate the measured values, each point represents the total cumulative sand fly abundance from the 12 trap-nights per treatment (i.e., 1 trap-night/house) and different species are indicated by different lines, see inset legend of Panel (**A**) for details.

Population dynamics of the five most abundant species can be observed in Figure 2. Figure 2A and 2B show, respectively, domicile abundance of fogged and control houses. Figure 2C shows peridomicile abundance of fogged houses, while Figure 2D shows peridomicile abundance of control houses. Inside the houses (domicile) the most abundant species were *Lu. trapidoi* and *Lu. gomezi* with a higher abundance in the control houses. In the peridomicile of the fogged houses *Lu. trapidoi* and *Lu. panamensis* were the most abundant species, while in the control houses, prior to the foggings, *Lu. triramula* was the most abundant species. Additional file 5: Table S2 presents the selection for the best model explaining the dynamics of the 5 most abundant species in our samples. All species had significant differences in their domicile and peridomicile abundance, all were sensitive to the fogging, *Lu. trapidoi* and *Lu. dysponeta* were sensitive to the synergistic (multiplicative) effects of monthly average daily rainfall and its standard deviation, meaning that these species could be sensitive not only to total rainfall but also to how variable the rainfall was during a time period, while *Lu. gomezi, Lu. panamensis* and *Lu. triramula* were sensitive to monthly S.D. of daily rainfall, in a non-linear relationship described by a second degree polynomial, which indicates these species thrive when there is sustained rain levels or when there is a high variability in the rain levels. In general terms all species were positively associated with rainfall, i.e., their abundance increased following increases in Rainfall. *Lu. triramula* was the most sensitive species to the insecticide fogging (Table 3), reducing its abundance by up to 91%, followed by *Lu. gomezi* (68%), *Lu. dysponeta* (51%), *Lu. panamensis* (40%). In contrast, *Lu. trapidoi* increased its abundance by 5% (Table 3).

**Table 3:** Parameter estimates for the negative binomial models explaining the abundance of selected Phlebotomine sand fly species in Trinidad de Las Minas, Capira, Panamá

**Parameter**	**Species**	**Proportional abundance change**	**Estimate**	**S.E.**	**z**	**Pr(>|z|)**
Domicile	*Lutzomyia trapidoi*	1 (563) ^¶^	6.333	0.794	7.980	<0.00001^x^
	*Lu. gomezi*	1 (38) ^¶^	3.647	0.220	16.586	<0.00001^x^
	*Lu. panamensis*	1 (7) ^¶^	2.010	0.212	9.467	<0.00001^x^
	*Lu. triramula*	1 (5) ^¶^	1.646	0.328	5.015	<0.00001^x^
	*Lu. dysponeta*	1 (16) ^¶^	2.752	0.745	3.694	0.000221^x^
Peridomicile	*Lu. trapidoi*	0.52	−0.646	0.306	−2.108	0.0350^x^
	*Lu. gomezi*	0.58	−0.551	0.278	−2	0.0454^x^
	*Lu. panamensis*	3.52	1.258	0.258	4.886	<0.00001^x^
	*Lu. triramula*	9.54	2.255	0.412	5.468	<0.00001^x^
	*Lu. dysponeta*	1.32	0.281	0.284	0.987	0.324
Fogged	*Lu.trapidoi*	1.05	0.0486	0.323	0.151	0.880
	*Lu. gomezi*	0.32	−1.128	0.292	−3.864	0.000112^x^
	*Lu. panamensis*	0.60	−0.515	0.271	−1.898	0.0576
	*Lu. triramula*	0.09	−2.381	0.452	−5.271	<0.00001^x^
	*Lu. dysponeta*	0.49	−0.711	0.306	−2.323	0.0201^x^
M-Rain	*Lu.trapidoi*	0.67	−0.397	0.142	−2.792	0.00524^x^
	*Lu. dysponeta*	0.66	−0.414	0.132	−3.125	0.00178^x^
S.D.-Rain	*Lu.trapidoi*	0.71	−0.337	0.0624	−5.401	<0.00001^x^
	*Lu. gomezi*	0.75	−0.289	0.100	−2.884	<0.00001^x^
	*Lu. panamensis*	0.76	−0.278	0.0936	−2.973	<0.00001^x^
	*Lu. triramula*	1.46	0.379	0.158	2.399	<0.00001^x^
	*Lu. dysponeta*	1.07	0.0669	0.0602	1.111	0.266
S.D.*M-Rain	*Lu.trapidoi*	1.03	0.0341	0.00859	3.973	<0.00001^x^
	*Lu. dysponeta*	1.01	0.0142	0.00812	1.751	0.0798
S.D.-Rain^2^	*Lu. gomezi*	1.01	0.0101	0.00386	2.630	0.00855^x^
	*Lu. panamensis*	1.01	0.0140	0.00358	3.928	<0.00001^x^
	*Lu. triramula*	0.99	−0.0106	0.00586	−1.807	0.0708

^x^Statistically Significant (P < 0.05). ^¶^the value inside parenthesis is the estimated abundance for the reference group, i.e., Control-Domicile. * indicates a synergistic (a.k.a. interaction) effect. The overdispersion parameter estimates (± S.E.) were 0.973 ± 0.210 for *Lu. trapidoi*, 1.182 ± 0.234 for *Lu. gomezi*, 1.487 ± 0.372 for *Lu. panamensis*, 0.623 ± 0.141 for *Lu. triramula* and 1.255 ± 0.302 for *Lu. dysponeta*.

Parameter indicates the different variables considered in the models for the selected sand fly species (see column with heading Species). Models considered the habitat (Domicile or Peridomicile), the Fogging (Fogged), and a second degree polynomial for the monthly S.D. of daily Rainfall (S.D.-Rain) for *Lutzomyia gomezi, Lu. panamensis* and *Lu. triramula;* and the interaction between S.D. and the mean (M-Rain) monthly daily rainfall for *Lu. trapidoi* and *Lu. dysponeta*.

### Spatial impacts of insecticide thermal fogging on Sand Fly abundance: the role of destitute housing

**Figure 3:**
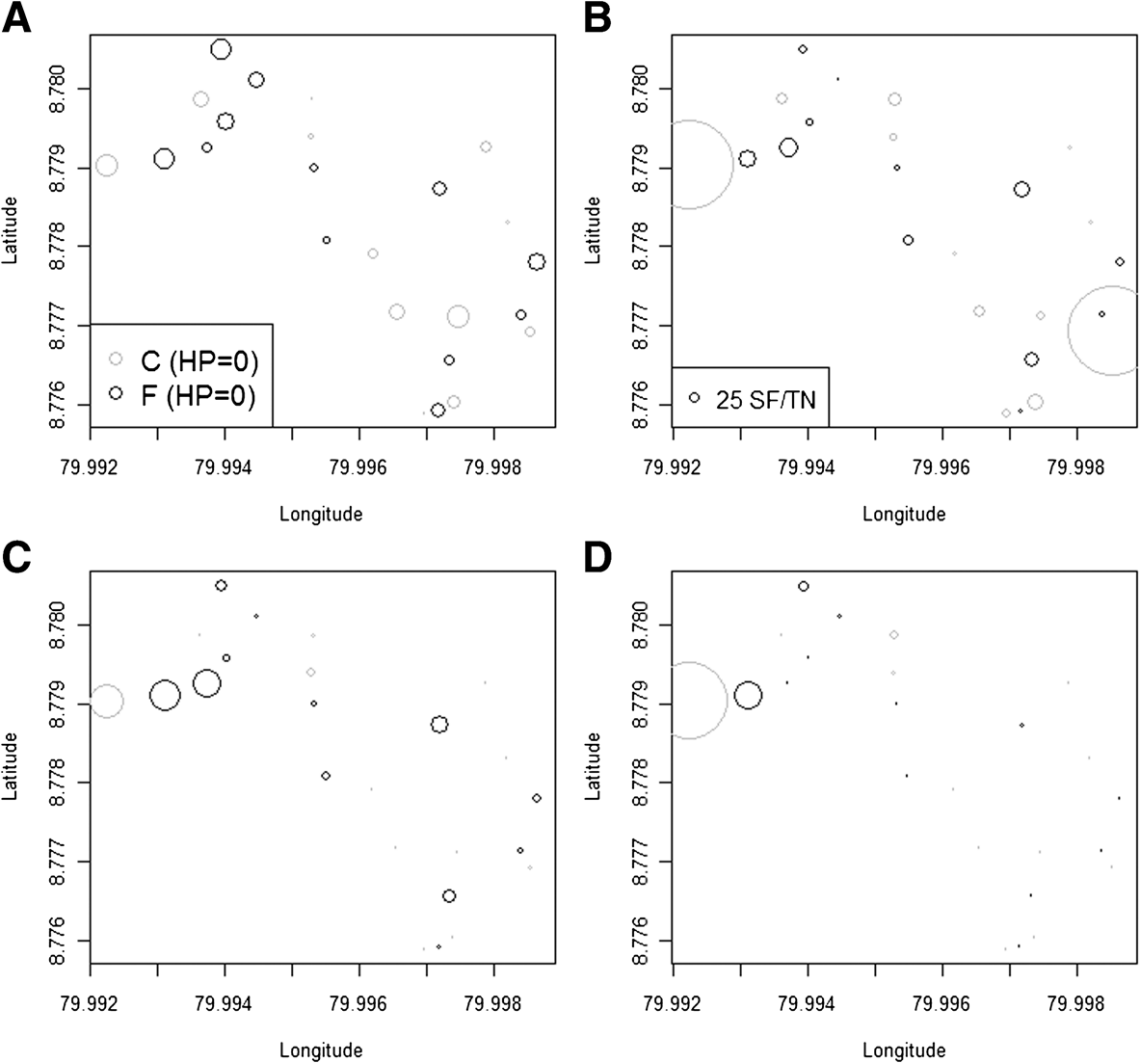
**Spatial patterns.** (**A**) Housing destituteness (HP) index. Circle size is proportional to the HP index, in the inset legend C (grey) stands for control and F (black) for fogged. Average sand fly density per house and trap night (B) Before the Fogging (**C**) After the 1st Fogging (**D**) After the 2nd Fogging. In (**B**), (**C**) and (**D**) circle size is proportional to the abundance of sand flies, see inset legend in (**B**) for reference (25 Sand Flies/trap night), and values were standardized by dividing the total cumulative abundance of each period by the number of trap nights in each period, i.e., 3, 4 and 5 respectively.

**Figure 4:**
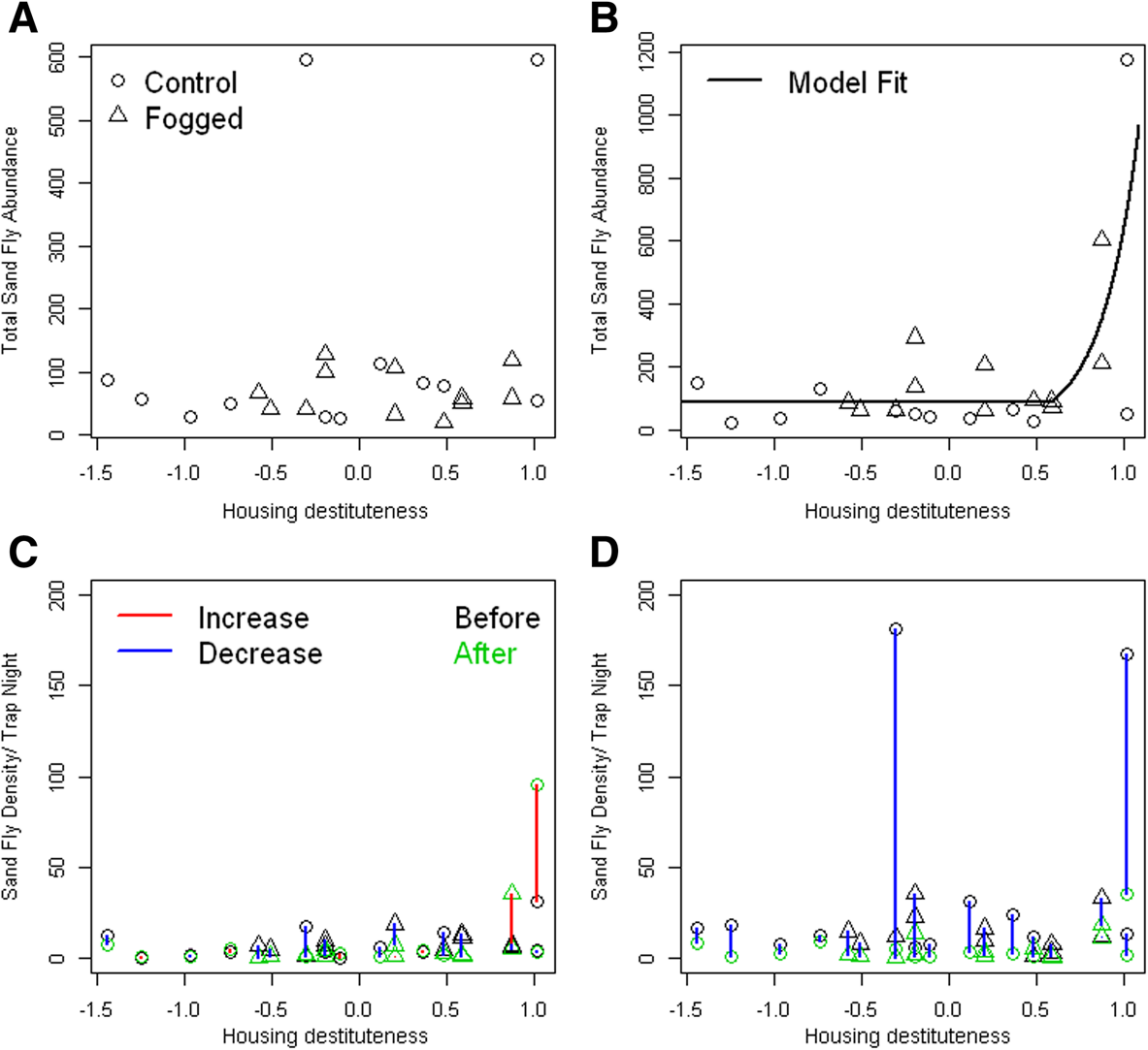
**Housing destituteness and sand fly abundance.** (**A**) Cumulative sand fly abundance before the fogging as a function of Housing Destituteness, HP (three traps nights) (**B**) Cumulative sand fly abundance for the nine traps nights that contained the two interventions as a function of HP. The solid black line is the fit from a negative binomial model with a breakpoint (**C**) Changes in domiciliary sand fly density per house and trap night as function of HP (**D**) Changes in peridomiciliary sand fly density per house and trap night as a function of HP. In panels (**A**) to (**D**) symbols indicate whether a house was intervened, triangles, or not, circles. In panels (**C**) and (**D**) black symbols represent the pre-intervention densities and green symbols post-intervention densities. To ease the tracking of changes in each house we joined the pre and post intervention densities with a line whose color is blue when sand fly density decreased and red when sand fly density increased. In panel (**C**), the solid black line represents Table 4 model fit.

Figure 3A shows the spatial patterns of housing destituteness, HP, which can be interpreted as a weighted average of the different components considered for the index (Additional file 6: Table S3). In general, high scores indicative of housing destituteness, i.e., features that could ease SF entrance into houses, resulted in a high HP index. Spatial patterns of SF infestation per trap the night before (Figure 3B) and after the first (Figure 3C) and second fogging (Figure 3D) in general suggest that, especially after the foggings, large infestations were positively associated with destitute housing conditions (Figures 3 and 4), but not with the peridomicile environment (Additional file 7: Figure S2, for index interpretation see Additional file 8: Table S4) the vegetation structure (Additional file 9: Figure S3, for index interpretation see Additional file 10: Table S5) or the animal abundance indices (Additional file 11: Figure S4, for index interpretation see Additional file 12: Table S6), or household residents (Additional file 11: Figure S4L). Nevertheless, the larger infestations before fogging were associated with a high peridomicile index (Additional file 7: Figure S2A), i.e., houses that had plenty of resting sites that were neither ornamental trees nor vegetable crops (Additional file 8: Table S4). In general, infestations seemed to be unrelated with whether the vegetation was tall, i.e.; high Vegetation Index, VI values, or ground cover i.e., low VI values (Additional file 10: Table S5, Additional file 9: Figure S3). Figure 4A shows the association between cumulative SF abundance prior to the fogging and HP. Figure 4B shows the association between cumulative SF abundance after the foggings and HP, which shows a clear pattern were the houses with the highest HP, on average, had the highest infestation. The curve shown in Figure 4B is the fit from a hockey stick regression (Table 4), which showed that, in general, after the foggings the highest infestations were observed in the most destitute houses, with sandflies increasing about ten times (11.6) for each 0.1 increase in HP above 0.586. For values below 0.586 infestation levels were homogeneous, with an average of 90 sand flies over 9 night-traps. The model presented in Table 4 had residuals that followed the assumptions of a NB-GLM and were not spatially correlated according to a Moran’s I test (*I* = 0.11; P > 0.18). The model in Table 4 was also selected from a larger ensemble of models that considered fogging, HP, PI, VI and the animal abundance indices monotonically associated with SF abundance, as well as, HP interactions with other variables (Additional file 13: Table S7). Finally, Figure 4C and 4D show the difference in the number of sand flies captured in the domicile and peridomicile, respectively, before and after the fogging of the studied households. Figure 4C shows that inside the houses, in general, SF density decreased or stayed the same, but for high HP, SF abundance tended to increase after the foggings. In contrast, Figure 4D shows that in the peridomicile environment SF abundance decreased independent of the degree of HP.

**Table 4:** Parameter estimates for a negative binomial model explaining post intervention heterogeneities in sand fly abundance across the households

**Parameter**	**Proportional abundance change**	**Estimate**	**S.E.**	**z**	**Pr(>|z|)**
Abundance	1 (90) ^¶^	4.501	0.159	28.354	<0.00001^x^
HP > 0.586	116	4.748	1.060	4.481	<0.00001^x^

^x^Statistically Significant (P < 0.05). ^¶^the value inside parenthesis is the estimated post intervention abundance.

Overdispersion parameter for the model was (± S.E.) 2.01 ± 0.55. The breakpoint for the Housing Destituteness Index (HP) was fixed at 0.586 and was estimated by a Brent optimization of the model Akaike Information Criterion.

## Discussion

The leishmaniases primarily affect poor people worldwide [39], but why poor populations are the most susceptible to CL infection in the New World requires a better understanding of how socio-economic human conditions alter CL transmission ecology [40,41]. SF abundance plays a major role in CL transmission, baseline results from our study area [45] and the whole República de Panamá [41,42,58] have shown a positive association between domiciliary vector abundance and CL human infections. Thus, our results suggest that housing quality, a socio-economic and ecological factor, could be a major entomological risk determinant for human infection with *Leishmania* spp parasites in rural Panamá. Our data clearly show how, beyond the transient impacts that insecticide applications could have on SF vector abundance, differences in housing quality may drive heterogeneities in SF house infestation, supporting suggestions from earlier SF insecticide control trials [23,32], which highlighted the role that poor housing quality could have on hampering SF control by insecticides. We found that houses with features that ease insect entrance, such as lack of anti-insect screening or with abundant holes, that were built with poor quality building materials (e.g., mud, which is more likely to generate places that can serve as resting sites for sand flies), or materials that can shorten the active lifespan of insecticides (e.g., wood or mud as opposed to concrete [23,26,32]), had the largest share of sand flies after the ITFs. In that sense, our results encourage further entomological research to understand exactly what factors make a household more susceptible to the infestation by sand flies. It will be ideal to devise simple housing modifications that can reduce the likelihood of SF household entry, as has been carried out with malaria vectors [59]. Also, a better understanding of the impact of peridomiciliary insecticide application on the recruitment of new adult sand flies into households requires more detailed study. As observed by Perich *et al.*[22], large scale barrier spraying can significantly reduce the number of sand flies entering an area. A similar decline in the number of sand flies inside the houses after an indoor plus peridomiciliary spraying was observed in Brazil [26] and Bolivia [25]. Nevertheless, it is not clear if there is an additional impact on SF abundance via a decrease in the recruitment of adults that may emerge from the peridomiciliary ground, given its potential to harbor SF larvae, either in tree trunks or the open ground [60-65] as the insecticide may also act upon SF larvae. For example, here we did not observe a rebound of peridomiciliary SF abundance after the 1st ITF, and this may be related to the recruitment of adults from larvae. However, it may also be related to eliminating a major source/refuge of adult sand flies with the ITFs [33].

Regarding the temporal impacts of deltamethrin on SF abundance in the fogged houses, insecticide action seemed to last up to 4 months, a shorter effect than what has been reported for higher concentrations of this insecticide when applied at 4-fold concentrations (~25 mg a.i.m^-2^), in residual spraying [25,26] or 2 to 10-fold concentrations (~12.5 to 60 mg a.i.m^-2^), when used on impregnated nets and curtains [28,29], where impacts seemed to last over 6 months. We think the difference may be a direct effect of insecticide concentration, which was significantly lower in our trial (~6 mg a.i.m^-2^). Also, unlike previous studies, we controlled for seasonality in SF abundance by incorporating rainfall in our temporal models, thus improving the ability to properly quantify the effect of the insecticide when compared with previous studies on SF insecticide control [20-35]. Our observations also raise some questions about the ideal frequency for insecticide application. For example, the only previous study in Panamá applied insecticides every 2 months based on practices for mosquito control [19], but our study suggests that such frequency might be too high given the low reactivity, i.e., ability of sand flies to respond to changes in their population abundance, which, for example, can be very high for mosquitoes [66]. Nonetheless, more detailed analysis of SF species population dynamics are necessary to better understand what would be an ideal frequency for insecticide application based on the dynamical properties of SF populations and not merely on insecticide bioassays.

A point deserving further attention is the impact of insecticide application on the community of SF species, especially when several vectors co-occur [67]. Even though *Lu. gomezi* and *Lu. panamensis*, two major vectors of *Leishmania panamensis*, the CL parasite in our study area, significantly reduced their abundance (more than 50% less abundant after the ITFs), *Lu. trapidoi*, traditionally the major CL vector in Panamá [42], increased its abundance by 5% after the ITFs. This observation maybe a byproduct of externally reducing the abundance of other dominant vectors and/or reflect some unaccounted variability of species seasonality [12]. However, it illustrates the need to more carefully look at vector control impacts, not only on dominant species but on the whole community of vectors [46,68,69]. We also need to acknowledge that a better handling of SF seasonality could have been possible in our models if we had access to more climatic variables, but only rainfall was tracked at the only weather station in our study area and we did not have resources to track meteorological information on our own.

Finally, our study has some limitations due to its relatively small scale. We are unable to tell whether there was a community wide impact of the patchy ITF on 12 of the 24 houses, and a better house pairing according to the destituteness was impossible given the lack of consent. However, we are confident our analysis shows that at the household level there is a reduction of SF abundance following ITF and that heterogeneities in SF reduction are related to housing quality. Still, scaling up SF insecticide control trials could be a desirable first step to improve CL control as it could allow a better understanding of the role of: (i) peridomiciliary (ii) patchy vs uniform and (iii) differential insecticide applications [33]. However, from our own experience, this is a major challenge given the neglected nature of CL and the lack of interested partners (funders, insecticide developers) on making larger scale trials possible or improving CL control.

## Conclusion

Our data clearly illustrate the importance of accounting for housing quality when evaluating insecticide control for leishmaniasis vectors and highlight the major role that destitute housing may have as a driving factor in the association between leishmaniasis and poverty.

## Abbreviations

ITF: Insecticide thermal fogging; SF: Sand fly; CL: Cutaneous leishmaniasis; HP: Housing destituteness index; PI: Peridomicile index; VI: Vegetation index; NB-GLM: Negative binomial generalized linear model

## Competing interests

All authors declare that they have no competing interests.

## Authors’ contributions

Conceived and designed the experiments: JEC, AZ, LFC. Performed the experiments: AZ, CR, AV, NG, JEC. Analyzed the data: LFC, JEC. Contributed reagents/materials/analysis tools: LFC, AZ, CR, AV, NG, JEC. Wrote the paper: LFC, JEC, AZ. All authors read and approved the final version of the manuscript.

## Supplementary Material

Additional file 1 **Protocol S1.** Power estimation [19,53,70] (PDF 45 kb) Click here for file

Additional file 2: Figure S1 Power Analysis (**A**) Assuming a 20% reduction on Sand Fly Abundance (**B**) Assuming a 50% reduction on Sand Fly Abundance. ITF = Insecticide Thermal Fogging. For further details see Protocol S1. Click here for file

Additional file 3 **Protocol S2.** Supplementary Methods [12,19,40,42,55,56,60-63,70-72]. Click here for file

Additional file 4: Table S1 Selection of the best negative binomial model explaining the abundance of all Phlebotomine Sand Flies in Trinidad de Las Minas, Capira, Panamá, following two foggings with deltamethrin [6 mg a.i.m^-2^]. Click here for file

Additional file 5: Table S2 Model selection for the best negative binomial models explaining the abundance of the five most abundant sand fly species in Trinidad de Las Minas, Capira District, Panamá, following two insecticide foggings with deltamethrin [6 mg active ingredient /m^2^]. Click here for file

Additional file 6: Table S3 Principal components analysis used to estimate the housing destituteness index. Click here for file

Additional file 7: Figure S2 Peridomicile index and sand fly abundance (**A**) Cumulative sand fly abundance before the fogging as a function of the peridomicile index, PI (three nights) (**B**) Cumulative sand fly abundance for the nine nights that contained the two interventions as a function of PI. The solid black line is the fit from a negative binomial model (**C**) Changes in domiciliary sand fly density per house and trap night as a function of PI (**D**) Changes in peridomiciliary sand fly density per house and trap night as function of PI. In panels (**A**) to (**D**) symbols indicate whether a house was intervened, triangles, or not, circles. In panels (**C**) and (**D**) black symbols represent the pre-intervention densities and green symbols post-intervention densities. To ease the tracking of changes in each house we joined the pre and post intervention densities with a line whose color is blue when sand fly density decreased and red when sand fly density increased. In panel B the dark line is the fit of an NB-GLM, where the intercept (± S.E.) is 4.94 ± 0.18 and the slope (± S.E.) is 0.67 ± 0.25. This means that for a PI of 0 there where ~140 sand flies and this number was doubled by each unit increase in PI. (TIFF 1986 kb) Click here for file

Additional file 8: Table S4 Principal components analysis used to estimate the peridomicile index. Click here for file

Additional file 9: Figure S3 **Vegetation index and sand fly abundance** (**A**) Cumulative sand fly abundance before the fogging as a function of Vegetation Index, VI (three nights) (**B**) Cumulative sand fly abundance for the nine nights that contained the two interventions as a function of VI. The solid black line is the fit from a negative binomial model (**C**) Changes in domiciliary sand fly density per house and trap night as function of VI (**D**) Changes in peridomiciliary sand fly density per house and trap night as a function of VI. In panels (A) to (D) symbols indicate whether a house was intervened, triangles, or not, circles. In panels (**C**) and (**D**) black symbols represent the pre-intervention densities and green symbols post-intervention densities. To ease the tracking of changes in each house we joined the pre and post intervention densities with a blue line when sand fly density decreased and red when sand fly density increased. Click here for file

Additional file 10: Table S5 Principal components analysis used to estimate the vegetation structure index. Click here for file

Additional file 11: Figure S4 Host abundance/richness and sand fly abundance. Cumulative sand fly abundance before the fogging (three nights) as a function of (**A**) Wild animal index (**B**) Domestic animal index (**C**) Wild and domestic animal index (**D**) Wild animal species richness (**E**) Domestic animal species richness (**F**) Household human density. Cumulative sand fly abundance after the foggings (nine nights) as a function of (**G**) Wild animal index (**H**) Domestic animal index (**I**) Wild and domestic animal index (J) Wild animal species richness (**K**) Domestic animal species richness (**L**) Household human density. Click here for file

Additional file 12: Table S6 Principal components analysis used to estimate the animal abundance indices. Click here for file

Additional file 13: Table S7 Model selection for the best negative binomial model explaining post-fogging sand fly abundance in the houses. Click here for file
