# Supplementary material for: Leishmaniasis sand fly vector density reduction is less marked in destitute housing after insecticide thermal fogging
Source: Parasit Vectors. 2013 Jun 6;6:164. doi: 10.1186/1756-3305-6-164 (PMC3693930; doi:10.1186/1756-3305-6-164)
Supplement: Additional file 1 — Protocol S1. Power estimation [19,53,70] (PDF 45 kb) [file 1756-3305-6-164-S1.pdf]

Protocol S1. Power Estimation

To estimate the power of our experimental design to capture insecticide thermal fogging (ITF) impacts on sand fly abundance, we employed the simulation approach described by Bolker [70]. In this approach, a statistical distribution for the response variable and expected means for the different treatments are assumed together with functional forms for possible covariates affecting the response variable. Thus, to compute the expected means of the treatments we made two assumptions: (1) HP light traps capture 3 times as many sand flies as common light traps [59] (2) fogged houses will see a decrease between 20% and 50 % in sand fly abundance following the fogging, as observed in the, to the best of our knowledge, only published study on sand fly control in Panama [19]. Since the number of sand flies per night trap in [19] was on average 10, then we assumed the mean value to be collected with a HP trap to be 30 (control), and 24 or 15 when, respectively, a 20% or 50% decrease in sand fly abundance is observed after ITF. We further assumed, in order to account for seasonality or spatial effects, the response was linearly associated with a predictor that increased linearly by two units and which was multiplied by a coefficient of 0.6 and $\alpha=0.05$, i.e., the probability of type I error was 5%. Finally, we assumed sand fly counts followed a Poisson distribution.
