# Supplementary figures and images for: Leishmaniasis sand fly vector density reduction is less marked in destitute housing after insecticide thermal fogging
Source: Parasit Vectors. 2013 Jun 6;6:164. doi: 10.1186/1756-3305-6-164 (PMC3693930; doi:10.1186/1756-3305-6-164)

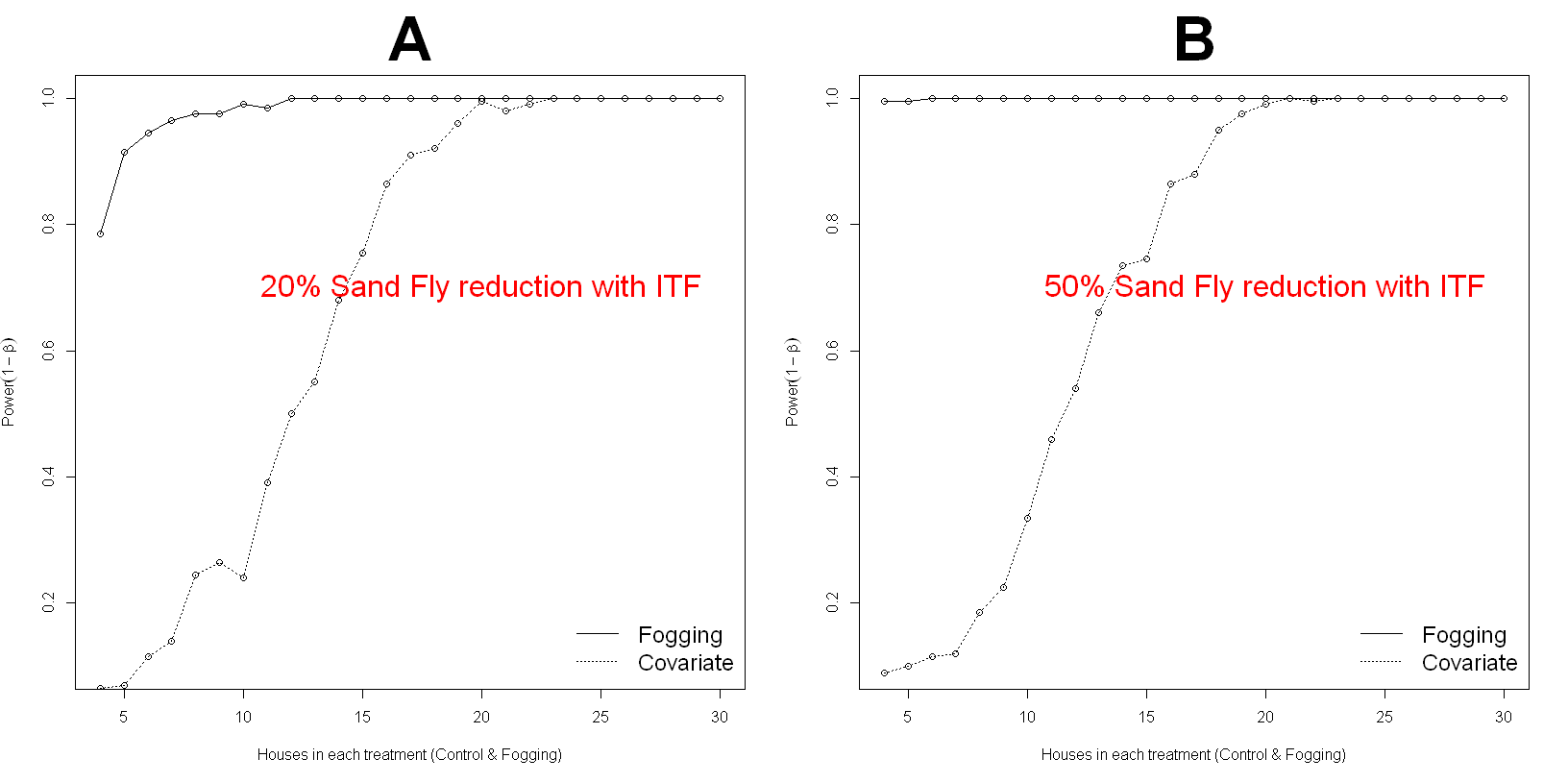

Supplement: Additional file 2: Figure S1 — Power Analysis (A) Assuming a 20% reduction on Sand Fly Abundance (B) Assuming a 50% reduction on Sand Fly Abundance. ITF = Insecticide Thermal Fogging. For further details see Protocol S1. [file 1756-3305-6-164-S2.tiff]

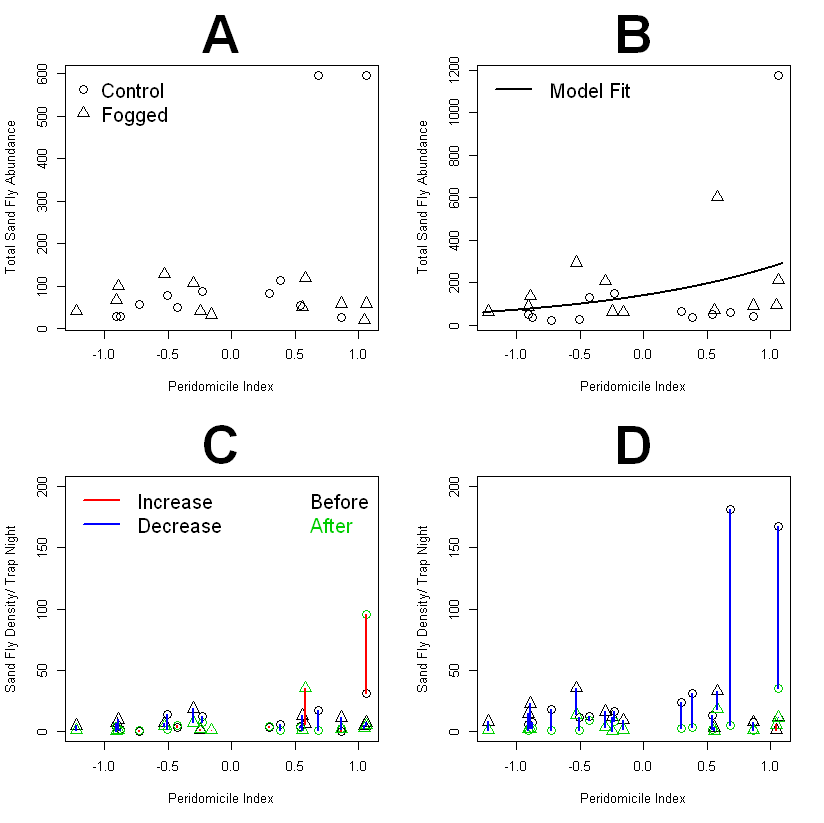

Supplement: Additional file 7: Figure S2 — Peridomicile index and sand fly abundance (A) Cumulative sand fly abundance before the fogging as a function of the peridomicile index, PI (three nights) (B) Cumulative sand fly abundance for the nine nights that contained the two interventions as a function of PI. The solid black line is the fit from a negative binomial model (C) Changes in domiciliary sand fly density per house and trap night as a function of PI (D) Changes in peridomiciliary sand fly density per house and trap night as function of PI. In panels (A) to (D) symbols indicate whether a house was intervened, triangles, or not, circles. In panels (C) and (D) black symbols represent the pre-intervention densities and green symbols post-intervention densities. To ease the tracking of changes in each house we joined the pre and post intervention densities with a line whose color is blue when sand fly density decreased and red when sand fly density increased. In panel B the dark line is the fit of an NB-GLM, where the intercept (± S.E.) is 4.94 ± 0.18 and the slope (± S.E.) is 0.67 ± 0.25. This means that for a PI of 0 there where ~140 sand flies and this number was doubled by each unit increase in PI. (TIFF 1986 kb) [file 1756-3305-6-164-S7.tiff]

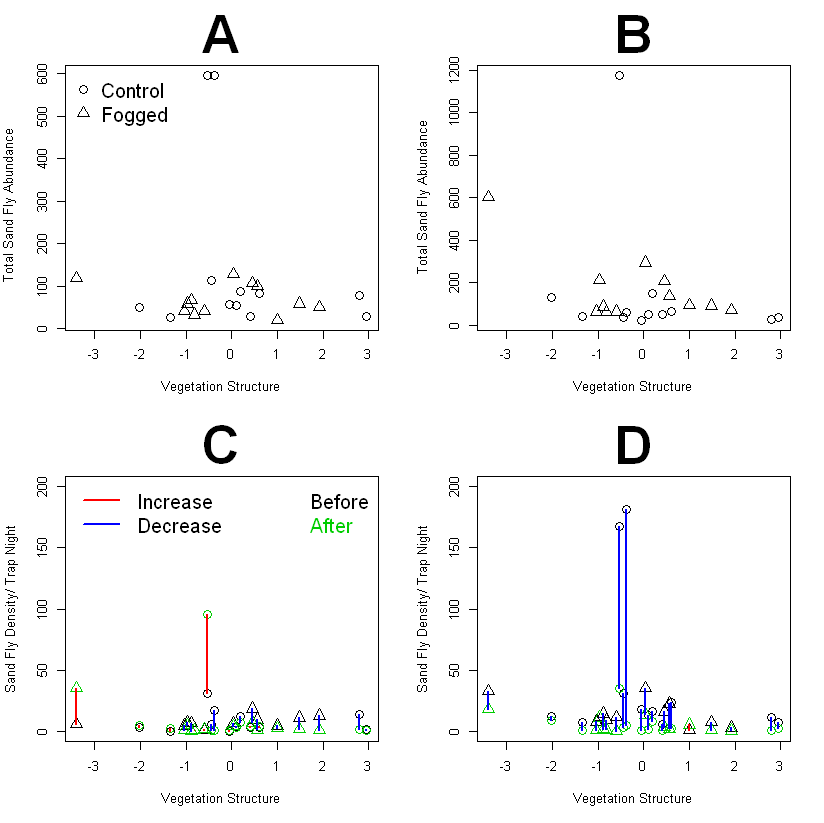

Supplement: Additional file 9: Figure S3 — Vegetation index and sand fly abundance (A) Cumulative sand fly abundance before the fogging as a function of Vegetation Index, VI (three nights) (B) Cumulative sand fly abundance for the nine nights that contained the two interventions as a function of VI. The solid black line is the fit from a negative binomial model (C) Changes in domiciliary sand fly density per house and trap night as function of VI (D) Changes in peridomiciliary sand fly density per house and trap night as a function of VI. In panels (A) to (D) symbols indicate whether a house was intervened, triangles, or not, circles. In panels (C) and (D) black symbols represent the pre-intervention densities and green symbols post-intervention densities. To ease the tracking of changes in each house we joined the pre and post intervention densities with a blue line when sand fly density decreased and red when sand fly density increased. [file 1756-3305-6-164-S9.tiff]

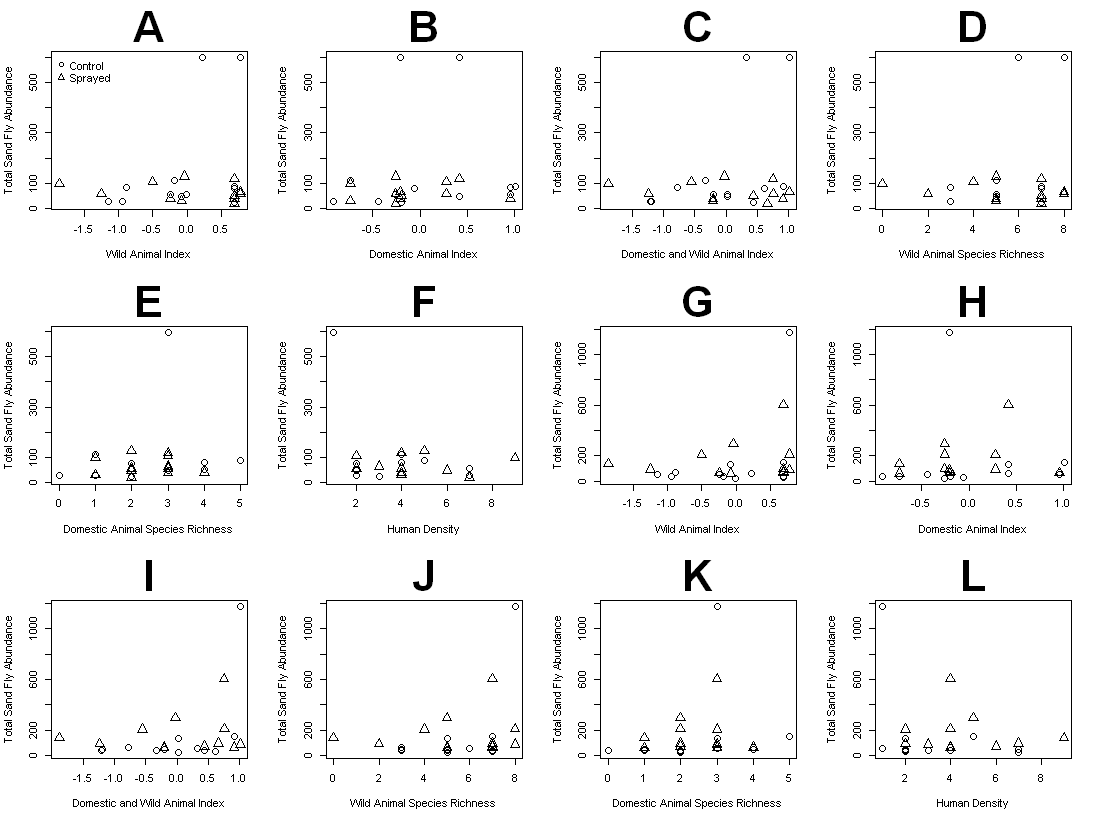

Supplement: Additional file 11: Figure S4 — Host abundance/richness and sand fly abundance. Cumulative sand fly abundance before the fogging (three nights) as a function of (A) Wild animal index (B) Domestic animal index (C) Wild and domestic animal index (D) Wild animal species richness (E) Domestic animal species richness (F) Household human density. Cumulative sand fly abundance after the foggings (nine nights) as a function of (G) Wild animal index (H) Domestic animal index (I) Wild and domestic animal index (J) Wild animal species richness (K) Domestic animal species richness (L) Household human density. [file 1756-3305-6-164-S11.tiff]
