# Supplementary material for: Leishmaniasis sand fly vector density reduction is less marked in destitute housing after insecticide thermal fogging
Source: Parasit Vectors. 2013 Jun 6;6:164. doi: 10.1186/1756-3305-6-164 (PMC3693930; doi:10.1186/1756-3305-6-164)
Supplement: Additional file 3 — Protocol S2. Supplementary Methods [12,19,40,42,55,56,60-63,70-72]. [file 1756-3305-6-164-S3.pdf]

Protocol S2. Supplementary methods

Housing destituteness (HP), peridomiciliary (PI) and vegetation structure (VI) and animal abundance and richness indices

For HP we assigned scores to the different materials used in the construction, where the smallest values represented conditions more likely to ease sand fly house infestation or materials more likely to resemble sand fly resting sites in nature: ceiling (1=thatched, 2=zinc); walls (1=wood, 2=concrete); floor (1=earthen, 2=wood or concrete); electricity (1=no, 2=yes); windows (1=without mesh, 2=with mesh, 3=no windows); crevices (1=yes, 2=no); complete walls (1=no, 2=yes); doors with holes (1=yes, 2=no). For the peridomicile index, PI, we assigned scores for the presence (1=yes, 0=no) of the following elements within the peridomicle, i.e., the 50 m radius around the houses: rubbish, water bodies (streams or pools), logs, stones, palms, vegetables, ornamental and fruit trees. All of these elements have been documented as adult sand fly resting places [12,19,42,55,60,61,62,63]. For the vegetation index, we measured canopy cover (tall trees, over 1.5 m), bush cover (trees below 1.5 m), ground cover (grasses and seedlings before 50 cm), canopy height (measured with a clinometer) and shade. For the vegetation structure sampling was done in a grid of 20m x 20m, measuring the vegetation structure in 4 randomly selected quadrats (10 m x 10 m) around the four edges of each house, a standard procedure in ecology [71]. For the domestic animal abundance index we counted all domestic animals belonging to each household. Domestic animals included chickens, dogs, cats, horses and parrots. We also recorded which wild animals have been seen by householders in the domiciliary/peridomiciliary environment. Wild animals included: sloths, opposums,

porcupines, voles, bats, squirrels, monkeys and birds. The animal abundance indices were estimated for: (i) wild, (ii) domestic and (iii) wild and domestic species. HP and PI were estimated by computing the first principal component of a variance/covariance matrix [56] for the variables considered in each index (see above). For VI and the animal abundance indices, we normalized (i.e., demeaned and divided by the S.D. each variable) all the VI [or animal abundance] variables before estimating the first principal component from the variance/covariance matrix of the VI [or animal abundance] variables. For VI [and animal abundance indices] the normalization was done to avoid artifacts in the principal components estimation, i.e.; the variables' heterogeneous variances can distort their patterns of association [56].

Hockey-stick models

We introduced breakpoints in predictors by transforming an independent variable using a breakpoint function with form:

$$f_L(x) = \begin{cases} b & x < b \\ 0 & otherwise \end{cases}$$
$$f_R(x) = \begin{cases} x & x > b \\ 0 & otherwise \end{cases} \qquad \text{(s1)}$$

where b is the breakpoint where the functions $f_L(x)$ and $f_R(x)$ join each other, and are used to separate the relationship between the dependent variable and the independent variables to the left and the right of the break point respectively [40,72]. Values for the breakpoints “b” were estimated a priori by minimizing the value for Akaike Information Criterion (AIC) of a function fitting the model while considering breakpoint values for the independent variables. For the minimization we employed the Nelder-Mead optimization algorithm [70].
