# Supplementary material for: Leishmaniasis sand fly vector density reduction is less marked in destitute housing after insecticide thermal fogging
Source: Parasit Vectors. 2013 Jun 6;6:164. doi: 10.1186/1756-3305-6-164 (PMC3693930; doi:10.1186/1756-3305-6-164)
Supplement: Additional file 4: Table S1 — Selection of the best negative binomial model explaining the abundance of all Phlebotomine Sand Flies in Trinidad de Las Minas, Capira, Panamá, following two foggings with deltamethrin [6 mg a.i.m-2]. [file 1756-3305-6-164-S4.pdf]

Table S1 Selection of the best negative binomial model explaining the abundance of all Phlebotomine Sand Flies in Trinidad de Las Minas, Capira, Panama following two foggings with deltamethrin [6 mg a.i.m^{-2}]. Feeding indicates the feeding preference (whether a species has been found feeding mainly on humans or not, habitat (domiciliary or peri-domiciliary), Fogging A (whether a house was fogged or was a control), Fogging B (whether a house was fogged, with different groups for the fogging event, or was a control) and monthly Rainfall (mean daily values, standard deviation of daily rainfall, S.D., and coefficient of variation= S.D. / mean), * indicates the interaction between factors. AIC stands for Akaike Information criterion and the minimum value is **bolded**.

Model Parameters	AIC
Feeding*FoggingA + Habitat*Feeding + Rainfall coefficient of variation	954.87
Feeding*FoggingA + Habitat*Feeding + Rainfall(mean)*Rainfall(S.D.)	953.57
Feeding*FoggingA + Habitat*Feeding + 2nd degree polynomial of Rainfall (mean)	955.67
**Feeding*FoggingA + Habitat*Feeding + 2nd degree polynomial of Rainfall (S.D.)**	**946.40**
Feeding*FoggingB + Habitat*Feeding + Rainfall coefficient of variation	958.86
Feeding*FoggingB + Habitat*Feeding + Rainfall(mean)*Rainfall(S.D.)	957.27
Feeding*FoggingB + Habitat*Feeding + 2nd degree polynomial of Rainfall (mean)	959.08
Feeding*FoggingB + Habitat*Feeding + 2nd degree polynomial of Rainfall (S.D.)	949.63
