# Supplementary material for: Leishmaniasis sand fly vector density reduction is less marked in destitute housing after insecticide thermal fogging
Source: Parasit Vectors. 2013 Jun 6;6:164. doi: 10.1186/1756-3305-6-164 (PMC3693930; doi:10.1186/1756-3305-6-164)
Supplement: Additional file 5: Table S2 — Model selection for the best negative binomial models explaining the abundance of the five most abundant sand fly species in Trinidad de Las Minas, Capira District, Panamá, following two insecticide foggings with deltamethrin [6 mg active ingredient /m2]. [file 1756-3305-6-164-S5.pdf]

Table S2 Model selection for the best negative binomial models explaining the abundance of the five most abundant sand fly species in Trinidad de Las Minas, Capira District, Panamá following two insecticide foggings with deltamethrin [6 mg active ingredient per m^2]. Model Parameters included: Habitat (Domicile and Peridomicile), Fogging (whether the counts came from a control or insecticide fogged house) and Rainfall monthly estimates for : daily coefficient of variation(CV), mean daily (M) and daily standard deviation (S.D.), * indicates the interaction between factors. AIC stands for Akaike Information criterion and the minimum value for each sand fly species is **bolded**.

Model Parameters	AIC				
	Lutzomyia trapidoi	*Lutzomyia gomezi*	*Lutzomyia panamensis*	*Lutzomyia triramula*	*Lutzomyia dysponeta*
Habitat+ Fogging + CV	388.6	398.4	367.9	311.1	310.9
Habitat+ Fogging + M*S.D.	**363.9**	399.7	350.3	305.9	**307.0**
Habitat+ Fogging + M^2	371.1	397.1	365.0	305.6	321.9
Habitat+ Fogging + $(S.D.)^2$	374.3	**393.3**	**346.7**	**304.5**	315.2
