# Supplementary material for: Leishmaniasis sand fly vector density reduction is less marked in destitute housing after insecticide thermal fogging
Source: Parasit Vectors. 2013 Jun 6;6:164. doi: 10.1186/1756-3305-6-164 (PMC3693930; doi:10.1186/1756-3305-6-164)
Supplement: Additional file 6: Table S3 — Principal components analysis used to estimate the housing destituteness index. [file 1756-3305-6-164-S6.pdf]

Table S3 Principal components analysis used to estimate the housing destituteness index. Variable indicates the variables and Comp.1, Comp. 2 and Comp. 3 indicate the loadings associated with each one of the three main principal components. The two bottom rows indicate the proportional variance and cumulative variance of the three main principal components.

Variable	Comp.1	Comp.2	Comp.3
Ceiling	0.150	0.263	-0.256
Wall Material	0.390	0	-0.146
Floor	0.384	0.567	-0.437
Electricity	0.282	0.552	0.653
Crevice	0.553	-0.327	-0.172
Complete Walls	0.188	0	0.49
Holes in Doors	0.507	-0.44	0.162
Proportion of Variance	0.33	0.25	0.16
Cumulative Proportion	0.33	0.58	0.74
