# Supplementary material for: Leishmaniasis sand fly vector density reduction is less marked in destitute housing after insecticide thermal fogging
Source: Parasit Vectors. 2013 Jun 6;6:164. doi: 10.1186/1756-3305-6-164 (PMC3693930; doi:10.1186/1756-3305-6-164)
Supplement: Additional file 8: Table S4 — Principal components analysis used to estimate the peridomicile index. [file 1756-3305-6-164-S8.pdf]

Table S4 Principal components analysis used to estimate the peridomicile index. Variable indicates the variables and Comp.1, Comp. 2 and Comp. 3 indicate the loadings associated with each one of the three main principal components. The two bottom rows indicate the proportional variance and cumulative variance of the three main principal components.

Variable	Comp.1	Comp.2	Comp.3
Rubbish	0.180	0.637	-0.405
Water Bodies	0.479	-0.171	0.414
Logs	0.543	0.107	0.295
Stones	0.567	0.178	-0.334
Palms	0.198	-0.487	-0.401
Ornamental	-0.183	0.504	0.413
Vegetales	-0.143	0.139	-0.365
Fruits	0.167	0.104	0
Proportion of Variance	0.32	0.21	0.13
Cumulative Proportion	0.32	0.53	0.66
