# Supplementary material for: Leishmaniasis sand fly vector density reduction is less marked in destitute housing after insecticide thermal fogging
Source: Parasit Vectors. 2013 Jun 6;6:164. doi: 10.1186/1756-3305-6-164 (PMC3693930; doi:10.1186/1756-3305-6-164)
Supplement: Additional file 10: Table S5 — Principal components analysis used to estimate the vegetation structure index. [file 1756-3305-6-164-S10.pdf]

Table S5 Principal components analysis used to estimate the vegetation structure index. Variable indicates the variables and Comp.1, Comp. 2 and Comp. 3 indicate the loadings associated with each one of the three main principal components. The two bottom rows indicate the proportional variance and cumulative variance of the three main principal components.

Variable	Comp.1	Comp.2	Comp.3
Canopy Cover	0.625	0	-0.258
Canopy Height	0.400	-0.611	-0.152
Ground Cover	-0.337	-0.181	-0.558
Bush Cover	0.205	0.424	-0.7
Shade	0.531	0	0.308
Proportion of Variance	0.34	0.22	0.17
Cumulative Proportion	0.34	0.56	0.73
