# Supplementary material for: Leishmaniasis sand fly vector density reduction is less marked in destitute housing after insecticide thermal fogging
Source: Parasit Vectors. 2013 Jun 6;6:164. doi: 10.1186/1756-3305-6-164 (PMC3693930; doi:10.1186/1756-3305-6-164)
Supplement: Additional file 12: Table S6 — Principal components analysis used to estimate the animal abundance indices. [file 1756-3305-6-164-S12.pdf]

Table S6 Principal components analysis used to estimate the animal abundance indices. Variable indicates the variables and Comp.1, Comp. 2 and Comp. 3 indicate the loadings associated with each one of the three main principal components. The two bottom rows indicate the proportional variance and cumulative variance of the three main principal components.

Index	Animals	Comp.1	Comp.2	Comp.3
Wild Animals	Opossums	0.374	0	0.297
	Squirrels	0.227	0	-0.168
	Voles	0.412	-0.555	-0.19
	Monkeys	0.463	0	-0.187
	Birds	0.322	-0.381	0.426
	Sloths	0.473	0.612	-0.137
	Porcupines	0	0	-0.732
	Bats	0.299	0.394	0.278
	Proportion of Variance	0.41	0.18	0.13
	Cumulative Proportion	0.41	0.59	0.72
Domestic Animals	Chickens	0.477	0.671	-0.153
	Dogs	0.18	0	0
	Cats	0.535	-0.556	-0.618
	Horses	0	0.485	-0.4
	Parrots	0.672	0	0.654
	Proportion of Variance	0.36	0.26	0.19
	Cumulative Proportion	0.36	0.62	0.81

Index	Animals	Comp.1	Comp.2	Comp.3
Domestic and Wild animals	Opossums	0.334	0	0
	Squirrels	0.203	0	0
	Voles	0.395	0	-0.438
	Monkeys	0.448	0	-0.132
	Birds	0.292	0	-0.222
	Sloths	0.431	0.23	0.528
	Porcupines	0.104	0.301	0
	Bats	0.293	-0.262	0.411
	Chickens	0.141	-0.258	0
	Dogs	0.158	-0.165	0.146
	Cats	0	-0.553	0.387
	Horses	0.253	0.109	0
	Parrots	0	-0.606	-0.329
	Proportion of Variance	0.28	0.16	0.14
	Cumulative Proportion	0.28	0.45	0.58
