# Supplementary material for: Leishmaniasis sand fly vector density reduction is less marked in destitute housing after insecticide thermal fogging
Source: Parasit Vectors. 2013 Jun 6;6:164. doi: 10.1186/1756-3305-6-164 (PMC3693930; doi:10.1186/1756-3305-6-164)
Supplement: Additional file 13: Table S7 — Model selection for the best negative binomial model explaining post-fogging sand fly abundance in the houses. [file 1756-3305-6-164-S13.pdf]

Table S7 Model selection for the best negative binomial model explaining post-fogging sand fly abundance in the houses. Parameters included an index for housing destituteness (Housing Destituteness), whether a house was fogged or not (Fogging). * indicates the interaction between factors. AIC stands for Akaike Information criterion and the minimum value for each sand fly species is **bolded**.

Model Parameters	AIC
Housing Destituteness * Fogging * Peridomicile index	284.31
Housing Destituteness * Fogging + Fogging * Peridomicile index	289.34
Housing Destituteness + Fogging + Peridomicile index	291.92
Housing Destituteness * Fogging	292.82
Housing Destituteness + Fogging	290.82
Peridomicile Index * Fogging	289.50
Peridomicile Index + Fogging	290.97
Peridomicile Index * Housing Destituteness	288.64
Peridomicile Index + Housing Destituteness	290.25
$(\text{Housing Destituteness})^2$	285.62
**Housing Destituteness with 1 breakpoint**	**278.29**
Housing Destituteness	288.82
$(\text{Peridomicile Index})^2$	291.98
Peridomicile Index with 1 breakpoint	290.49
Peridomicile Index	290.49
$(\text{Wild animal Index})^2$	291.85
Wild animal Index with 1 breakpoint	286.41
Wild animal Index	293.13
$(\text{Wild and domestic animal Index})^2$	288.70
Wild and domestic animal with 1 breakpoint	283.99
Wild and domestic animal	291.52
$(\text{Wild animal species richness})^2$	289.83
Wild animal species richness with 1 breakpoint	291.26
Wild animal species richness	292.66
